# Autoantibodies production and immunological abnormalities after bariatric surgery

**DOI:** 10.1016/j.jtauto.2019.100024

**Published:** 2019-11-09

**Authors:** Gabriel J. Tobón, Fabio E. Ospina, Juan Pablo Suso, Iván Posso-Osorio, Andrés F. Echeverri, Evelyn Muñoz-Buitrón, Javier-Darío Martínez, Gloria-Lucía Castaño, Andrés Agualimpia, Fabio Bonilla-Abadía, Evelyn Dorado, Carlos A. Cañas

**Affiliations:** a Grupo de Investigación en Reumatologia, Autoinmunidad y Medicina Traslacional (GIRAT), Fundación Valle Del Lili and Universidad Icesi, Cali, Colombia; b Laboratory of Immunology, Fundación Valle Del Lili, Cali, Colombia; c Center for Clinical Research, Fundación Valle Del Lili, Cali, Colombia; d Department of Surgery, Fundación Valle del Lili, Cali, Colombia

**Keywords:** Bariatric surgery, Autoimmune diseases, Immunology

## Abstract

**Objective:**

Bariatric surgery is a widely used procedure for the treatment of obesity. Our aim is to describe the main immunological changes in patients who undergo bariatric surgery.

**Methods:**

A prospective study was conducted within a cohort of patients undergoing bariatric surgery and without previous evidence of systemic or organ-specific autoimmune diseases in whom 3 blood samples were collected – one day before surgery (Time 0), and 5 (Time 1) and 10 months (Time 2) after surgery.

**Results:**

Thirty four obese patients underwent surgery (Time 0):30(88.24%) were women, mean age 38.3 years. When comparing Time 0 and Time 2, there were statistically significant changes in CD4+T cell count, with an increase from 1074/mL(IQR:860–1316) to 1217.5/mL(IQR:838–1510),*p* = 0.0002. The CD4/CD8 ratio increased from 2.2(IQR: 1.7–2.7) to 2.4(1.8–2.8), *p* = 0.0001. As for humoral variables, the C3 fraction of complement decreased from 164 ± 40.6 mg/dL to 112.4 ± 31.4 mg/dL(*p* < 0.001) and C4 decreased from 29.3 ± 10.1 mg/dL to 22.5 ± 7.1(*p* = 0.0009) at Time 2. Four patients with negative ANAs at baseline, showed positive ANAs at Time 2.One patient developed anti-citrullinated peptide antibodies >200 IU/mL at Time 2.

**Conclusions:**

Patients undergoing bariatric surgery show immunological changes which might eventually lead to develop an autoimmune disease.

## Highlights

• Bariatric surgery is beneficial in treatment of obesity, although little is known about impact of significant weight loss in the immune system.
• Bariatric surgery may induce antinuclear antibodies positivity.
• Bariatric surgery may induce changes in T lymphocytes population.

## 1 Introduction

Bariatric surgery (gastric bypass or gastric sleeve) is a widely used procedure worldwide for the treatment of obesity, for achieving adequate weight loss and for managing obesity-related comorbidities [1].

However, the role of weight loss on the immune system is still controversial and even more when weight loss can be massive and fast. In a previous article by our group, we described the development of autoimmune diseases following weight loss associated with bariatric surgery [2]. In addition, some cases reported in the world literature have shown that a marked decrease in body mass index (BMI) might possibly trigger autoimmunity-related rheumatologic syndromes and metabolic processes resulting mainly from rapid weight loss [2]. Currently, the effects of bariatric surgery on the immune system, including the different T and B cell subpopulations, autoantibodies, complement and markers of inflammatory activity are unknown.

In this study, we prospectively evaluated 34 obese patients with no clinical or laboratory evidence of autoimmune diseases in whom bariatric surgery was performed. We conducted thorough biochemical and hematological tests, autoantibodies panel and peripheral blood distribution of T and B cells at baseline and in a two-step follow-up after the procedure (5 and 10 months on average) to evaluate any possible changes in the immunological field and to establish any possible mechanisms related to the onset of systemic autoimmune diseases triggered by weight loss.

## 2 Methods

Between September 2014 and December 2015, a prospective cohort study of patients undergoing bariatric surgery for treating obesity and without previous clinical or laboratory evidence of systemic or organ-specific autoimmune diseases was performed. In these patients, 3 serial blood samples were collected at baseline and 5 and 10 months after the procedure. Inclusion criteria were obese patients over 18 years, candidates for surgical management (sleeve or gastric bypass), who signed an informed consent. Patients with autoimmune rheumatologic diseases, malignancies and immunosuppression were excluded.

### 2.1 Study design

Prospective descriptive study, where patients were evaluated by a rheumatologist before surgery (Time 0) for a complete medical history and physical examination with anthropometric assessment, and peripheral blood samples were collected for determination of T and B cell peripheral distribution by flow cytometry (processed within the first 6 h of collection) and serum storage at −80 °C for processing ANAs, C3, C4, IgG and IgM anti-cardiolipins, anti-citrullinated peptide antibodies (ACPA), rheumatoid factor (RF), leptin and adiponectin. These parameters were evaluated at two additional timepoints [on average 5 (Time 2) and 10 months (Time 3) after surgery]. All parameters were titrated to determine any possible immunological changes at follow-up. The study protocol was approved by the Ethics Committee, and all patients signed an informed consent.

### 2.2 Laboratory determinations

C3 and C4 levels were assessed by immunoturbidimetry using the Roche Cobas® e501 analyzer (Tokyo, Japan) with normal values between 90 and 140 mg/dL and 10–40 mg/dL, respectively. The presence of anti-nuclear antibodies (ANAs) was determined by indirect immunofluorescence (IFI) technique using the Inova Kit Hep2 (San Diego, USA). Anti-cardiolipin IgM, IgG and ACPA titers were determined by ELISA using the Orgentec® kit (Mainz, Germany). Rheumatoid factor was determined by immunoturbidimetry using the Roche® Cobas analyzer (Tokyo, Japan). Peripheral blood B and T cell phenotyping was performed by flow cytometry using CD3^+^ markers for T cell separation, CD3^+^ and CD4^+^ for helper T cells, CD3^+^ and CD8^+^ for cytotoxic lymphocytes, CD19 ^+^ for B cells using the Beckman Coulter® tetrachrome CD45-FITC/CD4-RD1/CD8-ECD/CD3-PC5 CYTO-STAT kit (Fullerton, USA). Adipokines (leptin and adiponectin) were measured by solid-phase enzyme-amplified sensitivity immunoassay on microplates using the Inova® DIAsource leptin-EASIA kit (Louvain-la Neuve, Belgium) and the Inova® DIAsource adiponectin ELISA kit (Nivelles, Belgium), respectively.

### 2.3 Statistical analysis

A descriptive exploratory analysis was performed. Continuous variables were expressed as means ± SD, median and interquartile ranges, and were analyzed using a paired *t*-test or Wilcoxon signed-rank test, depending on the achievement of normality assumptions. For data analysis, the STATA 12.1 program was used. As for comparisons, *p* values < 0.05 were considered significant. The Pearson’s correlation coefficient was used to determine correlation between continuous variables.

## 3 Results

A total of 34 patients who met the inclusion criteria were included in the study (Table 1), of which 30 were women (88.2%). The mean age at surgery (Time 0) was 38.3 ± 11.57 years. As for medical history, 1 had no comorbidities (2.94%), 13 had hypertension (38.24%), 5 patients had a history of pseudotumor cerebri (14.71%), 18 had hyperlipidemia (52.94%) and 2 had a history of previously treated cancer (5.88%). The second assessment (Time 1) was on average at 174.78 ± 53 days; at this timepoint, 2 patients were lost to follow-up. The third evaluation (Time 2) was performed on average at 305.4 ± 75.7 days. At Time 2, 2 more patients were lost to follow-up.

**Table 1:** Patient characteristics, medical history and baseline symptoms.

Variable	N (%)
Female patients	30 (88.2%)
Average age at surgery	38.3 years
**Medical history**	
Hypertension	13 (38.24%)
Hyperlipidemia	18 (52.94%)
Pseudotumor cerebri	5 (14.71%)
Previously treated cancer	2 (5.88%)
Knee osteoarthritis	15 (46.88%)
**Rheumatologic Symptoms**	
Dryness symptoms	0 (0%)
Joint pain	0 joints (32.35%), n = 11 1 joint (26.47%), n = 11 2 joints (23.53%), n = 8 3 or more joints (17.65%), n = 6

*Joint symptoms:* Presence of joint symptoms were evaluated prior to surgery. Eleven patients (32.35%) had no joint symptoms, 9 patients had pain in 1 joint (26.47%), 8 patients had pain in 2 joints (23.53%) and 6 patients had pain in more than 2 joints (17.65%). In all patients, the pain showed mechanical characteristics with no evidence of inflammation or morning stiffness. During the Time 1, 23 patients had no joint symptoms (67.65%), 8 patients had pain in 1 joint (23.53%), and 1 patient had pain in 2 joints (2.94%). At Time 2, 10 months after surgery, 18 patients were asymptomatic for joint involvement. Inflammatory joint involvement was observed in no patients.

*Dryness symptoms:* Prior to surgery, no patients reported either subjective or objective dryness symptoms (dry eye or dry mouth). At time 1, 5 patients (14.71%) had dryness symptoms (xerophthalmia and xerostomia in all 5 patients and xerosis in 2 of them). At Time 2, this number decreased to 2 patients who had dry mouth only (5.88%).

*Other symptoms:* As for other symptoms, all patients were asymptomatic prior to surgery. During the first follow-up period, 13 patients (38.24%) reported increased hair loss, and one patient (2.94%) had neurological manifestations compatible with Guillain-Barre syndrome.

*Cellular immunological parameters:* B- and T-cell phenotype was assessed in peripheral blood samples at all three different times. Table 2 and Table 3 show the numeric and percentage results for each of the three times.

**Table 2:** Baseline (Time 0) versus follow-up at 5 months (Time 1) comparison.

N = 32	Baseline (Time 0)	Time 1	*p*-value
% LTCD3^b^	69.7 (66.2–75.1)	70.4 (66.1–72.5)	0.5188
%LTCD4^a^	44.5 ± 8.3	45.2 ± 7.1	0.316
% LT CD8^a^	21.7 ± 7.2	21 ± 6.4	0.0555
CD4 count^b^	1142 (890.5–1337)	1033.5 (903.5–1361)	0.8811
CD8 count^a^	564.6 ± 328.6	558.6 ± 300.6	0.7967
CD4/CD8 ratio^b^	2.2 (1.7–2.6)	2.3 (1.7–2.7)	0.0628
% LB CD19^b^	16.3 (12.9–20.3)	16.4 (12.7–19.5)	0.6808
% LB CD20^b^	16.5 (13–20.2)	16.2 (12.6–18.4)	0.489
CD19 count^b^	415.5 (278.5–570)	356 (283.5–544)	0.8959
CD20 count^b^	407 (275–574.5)	351 (276.5–543.5)	0.9925
C3^a^	164.7 ± 39.1	152.8 ± 23.9	0.0952
C4^a^	28.4 ± 10.4	30.5 ± 8.1	0.0714
Leptin	45.7 ±9.14	23.5 ±12.6	<0.001
Adiponectin^a^	21.3 (17.8–34.1)	33.5 (23.85–50.05)	< 0.0001
Anticardiolipin IgG antibodies b	7.4 (6.1–10.4)	6.4 (5.1–9.4)	**0.0125**
Anticardiolipin IgM antibodies b	0.95 (0.75–1.4)	1.15 (0.8–1.5)	0.1711
Rheumatoid factor^a^	5.7 ± 1.7	5.1 ± 1.8	0.0283

a Mean ± SD.

b Median (IQR).

**Table 3:** Baseline (Time 0) versus follow-up at 10 months (Time 2) comparison.

N = 30	Time 0	Time 2	*p*-value
% LTCD3*	69.1 ± 7.7	69 ± 6.5	*0.9676*
%LTCD4*	44.6 ± 8.3	45.8 ± 7.4	***0.0179***
% LT CD8**	21 (18–24.9)	20.1 (16.9–23.2)	***0.0002***
CD4 count **	1074 (860–1316)	1217.5 (838–1510)	***0.0016***
CD8 count **	494 (355–634)	502.5 (378–632)	*0.3876*
CD4/CD8 ratio **	2.2 (1.7–2.7)	2.4 (1.8–2.8)	***0.0001***
% LB CD19*	16.7 ± 6.1	16.5 ± 5.2	*0.5881*
% LB CD20**	15.9 (12.8–20)	15.6 (12.8–19.1)	*0.2989*
CD19 count*	416 ± 202.3	448.8 ± 187.3	*0.1808*
CD20 **	404 (272–511)	426 (293–588)	*0.2667*
C3*	164 ± 40.6	112.4 ± 31.4	***<0.001***
C4*	29.3 ± 10.1	22.5 ± 7.1	***0.0009***
Leptin	44.12 ± 9.75	22.49 ± 9.7	***<0.001***
Adiponectin*	29.2 ± 20.17	40.8 ± 23.4	***0.042***
Anticardiolipin IgG antibodies**	7 (6–10.5)	3 (2.6–5.2)	***<0.001***
Anticardiolipin IgM antibodies **	0.95 (0.7–1.4)	0.95 (0.8–1.4)	0.4601
Rheumatoid factor*	5.5 ± 1.7	3.6 ± 1.7	<0.001

*Mean ​± ​SD, ** Median (IQR), *Paired *t*-test, ** Wilcoxon signed-rank test.

None of the evaluated parameters showed statistically significant changes when comparing Time 0 versus Time 1 data. When comparing Time 0 versus Time 2, several phenotypic changes were evidenced. For instance, CD4^+^ T cell count increased from 1074/mL (IQR: 860–1316) to 1217.5/mL (IQR: 838–1510), *p* = 0002; while the percentage of CD4^+^ T cells increased from 44.6 ± 8.3 to 45.8 ± 7.4 with a *p* value of 0.0179. Although an increased CD8^+^ lymphocytes count from 494/mL (IQR: 355–634) to 502.5/mL (IQR: 378–632) was evidenced, this result was not significant (*p* = 0.38). The CD4/CD8 ratio increased from 2.2 (IQR: 1.7–2.7) to 2.4 (1.8–2.8), *p* = 0.0001. The remaining lymphocytic populations (CD19 and CD20) showed no changes at Time 1 and Time 2.

*Serum immunological parameters:* Several serum values also showed statistically significant changes. Complement C3 decreased from 164 ± 40.6 mg/dL to 112.4 ± 31.4 mg/dL (*p*-value <0.001) and C4 decreased from 29.3 ± 10.1 mg/dL to 22.5 ± 7.1 mg/dL (*p* = 0.0009 at Time 2); however, Time 0 versus Time 1 comparisons were not statistically significant.

When comparing Time 0 *vs.* Time 1, leptin decreased from 45.7 ± 9.14 ng/mL to 23.5 ± 12.6 ng/mL (*p*-value <0.001) and, when comparing Time 0 versus Time 2, leptin decreased from 44.12 ± 9.75 ng/mL to 22.49 ± 9.7 ng/mL (*p*-value of <0.001). As for adiponectin, when comparing baseline values versus Time 1, a significant increase from 21.3 (17.8–34.1) ng/mL to 33.5 (23.85–50.05) ng/mL (*p* < 0.0001) was evidenced. When comparing Time 0 versus Time 2, changes were also statistically significant, with 29.2 ± 20.17 ng/mL for Time 0 and 40.8 ± 23.4 for Time 2 (*p* = 0.042).

*Autoantibodies:* At Time 0, all patients were negative for ANAs. At Time 2, 4 patients (11.76%) increased ANA concentrations: 3 cases with homogeneous pattern (1:80 titer) and one patient with centriole pattern (1:160 titer).

ACPA antibodies at Time 0 were on average 8 ± 3.2 IU/mL (normal up to 20 IU/mL); no changes were observed at Time 1, with values of 8 ± 4.2 (*p* = 0.8271), or Time 2: 7.8 (6.6–9.7) (*p* = 0.0916). Despite the lack of changes in ACPA titers, one patient developed positive anti-citrullinated peptide antibodies (ACPA) at the upper limit of 20 IU/mL (reference values <20 IU/mL) at Time 0, with a 10-fold titer increase (200 IU/mL) at Time 2.

IgG anticardiolipin antibodies showed a statistically significant change when comparing Time 0 versus Time 1, decreasing from 7.4 (6.1–10.4) GPL to 6.4 (5.1–9.4) with a *p* value of 0.0125; this difference was also observed when comparing Time 0 versus Time 2, with a decrease from 7 (6–10.5) to 3 (2.6–5.2) (*p* < 0.001).

IgM anticardiolipin antibodies showed no statistically significant changes when comparing all times. When evaluating RF, there were statistically significant differences, with values of 5.7 ± 1.7 IU/mL at Time 0 and a significant decrease to 5.1 ± 1.8 at Time 1, with a *p* value of 0.0283. This decrease was also statistically significant when comparing Time 0 versus Time 2, with a decrease of 5.5 ± 1.7 at Time 0 and values of 3.6 ± 1.7 at Time 2, with a *p*-value of <0.01.

Then, we evaluated the correlation between percentage loss weight and biological variables at 10 months using the Pearson’s coefficient test. None of the evaluated variables showed a correlation: CD4 lymphocyte count (r: −0.0091; *p*-value: 0.962), CD8 lymphocyte count (r: 0.0822; *p*-value: 0.666), complement C3 (r: −0.2073; *p*-value: 0.2716), complement C4 (r: 0.0697; *p*-value: 0.7145), adiponectin (r: 0.16442) and leptin (r: 0.1733; *p*-value: 0.4073).

## 4 Discussion

The valuable effects of bariatric surgery have been widely described. These include, but are not limited to, improvement in dyslipidemia, decrease in cardiovascular risk, and improvement in hypertension, osteoarthritis, and diabetes mellitus [3]. However, the effects of these types of surgeries on the immune system are not well known. In a previous article published by our group, we documented the development of systemic autoimmune diseases [rheumatoid arthritis (RA) and systemic lupus erythematosus (SLE)] following bariatric procedures indicated for the management of obesity [2].

The explanation for these cases may rely on the context of rapid weight loss in genetically susceptible individuals leading to the development of autoimmune conditions or to subclinical underlying immune disorders. Due to this clinical observation, this prospective study was designed to determine the immunological changes associated with bariatric surgery. Although changes in some cell populations, mainly T cells, and in complement levels and adipokines were observed in addition to the development of autoantibodies, no subjects developed any clearly established disease during follow-up.

Leptin is an adipokine with multiple functions on the immune system, including inhibition of NK lymphocytes and stimulation of monocyte proliferation and activation [4]. In this study, the decreased levels of this hormone may be explained by the lower postoperative percentage of body fat in these patients.

In contrast, adiponectin is an anti-inflammatory adipokine with multiple effects on the immune system, particularly those affecting synovial fibroblasts where adiponectin induces IL-6 and IL-10 formation [4]. This study showed a significant increase in this hormone, which is consistent with previous literature reports describing adiponectin suppression in obese patients because of a down-regulation of the same adipose tissue producing it [5]. Therefore, a decrease in body fat percentage increases adiponectin levels.

In the study by Illán-Gómez et al. [6], patients undergoing bariatric surgery were shown to have high adiponectin levels compared to baseline values up to 1 year after surgery as well as decreased IL-6 and C-reactive protein levels, which demonstrated improvement in the inflammatory pattern of these patients.

In a study carried out in South Korea by Kyoung Kim et al. [7], serum levels of other important adipokines such as BMP-4 (Bone Morphogenetic Protein 4) and PAI 1 (Plasminogen Activator Inhibitor-1) were decreased after bariatric surgery, showing a shift to an anti-inflammatory state.

All these findings may suggest that bariatric surgery is beneficial for inflammatory states and might protect against the development of autoimmune diseases. However, our findings are contradictory because they show, in the first place, that some individuals may develop autoimmune diseases after losing weight, and secondly, that several immunological changes occur.

As for the cellular component of the immune system, we observed significant changes in CD4^+^ lymphocyte counts, changes which have not been previously reported in the literature. These changes may be explained by the suppressive effects of leptin on CD4^+^ T cells [8] and by the findings that a decrease in this hormone may increase CD4^+^ levels.

In this study, complement levels showed a statistically significant decrease when comparing Time 0 versus Time 2. This decrease may be due to either low production or high consumption. Since no patients showed clinical manifestations of autoimmune diseases, the latter option appears to be less probable. As a result, we hypothesize that this may be due to a lower synthesis of complement in the liver because of the decreased inflammatory state occurring in obesity. This should be verified by evaluating the functional role of complement (CH50, CH100).

In terms of positive autoantibody tests, 4 patients (11.76%) were found to develop positive ANAs, which may suggest that, in certain cases, weight loss is associated with immune disorders resulting in the development of antibodies. As for ACPAs, although no significant average changes were observed, one patient had a 10-fold increase in its levels, which could be explained by a genetic predisposition that is necessary for changes to occur in addition to an environmental component.

In this study, the role of autoantibodies, complement, cytokines and cellular changes for the future development of autoimmune diseases is unclear and should be assessed during the prospective follow-up of our patients. However, based on the article by Arbuckle et al. [9] that showed that patients with SLE had autoantibodies for approximately 6 years prior to the clinical diagnosis of the disease, we should hypothesize that patients with immunological changes might have a higher risk for developing autoimmune diseases.

This study provides information that has been scarcely described in the literature, about the possible effects on the immune system of bariatric surgery and rapid weight loss. On the other hand, some publications have shown the benefit of weight loss on clinical and laboratory outcomes in patients with established autoimmune or inflammatory diseases undergoing bariatric surgery. For example, a retrospective study published by Egeberg et al. [10] showed that patients undergoing gastric bypass had a lower risk for developing psoriatic arthritis and that those already diagnosed with the disease had significant improvement in disease activity. These changes were not significant for patients undergoing “gastric banding”. Although the reasons for this difference are unclear, there may be a greater difference in the secretion of hormones able to modulate inflammation in patients with gastric bypass as compared to those with gastric ligation. In our study, no difference was observed in patients undergoing either procedure.

In the context of patients with established autoimmune diseases and concomitant obesity, the effects of obesity on the course of inflammatory diseases have been demonstrated. For example, a study showed that disease remission in obese patients with RA was less likely than in patients with normal weight [11].

A study by Corcelles et al. [12] in patients with SLE undergoing bariatric surgery showed that, despite a higher risk of short-term postoperative complications such as infection, long-term complications resulted in lower requirements for immunosuppression.

A retrospective study conducted in the United States by Sparks et al. [13] showed favorable outcomes after bariatric surgery in patients with RA. At the time of surgery, 57% of patients had moderate to high disease activity; 12 months after surgery, the percentage decreased to 6%, with a *p* value of 0.001. In addition, at surgery, 26% of patients were on remission; at 12 months, this percentage had a statistically significant increase to 74%. Likewise, C-reactive protein, erythrocyte sedimentation rate and use of DMARDs showed a significant decrease.

In conclusion, our study shows that obese patients undergoing bariatric surgery have subsequent immunological changes. Although the development or lack of development of autoimmune diseases is not predictable, awareness about this possibility is mandatory. Further studies are necessary to define any predisposing factors for the development of clinically significant immunological changes.

## Funding

The study was funded by 10.13039/501100002411Universidad Icesi and 10.13039/501100003402Fundación Valle del Lili.

## Declaration of Competing interest

The authors declare they have no conflicts of interest.
